# Accelerated Generation of Selfed Pure Line Plants for Gene Identification and Crop Breeding

**DOI:** 10.3389/fpls.2017.01786

**Published:** 2017-10-24

**Authors:** Guijun Yan, Hui Liu, Haibo Wang, Zhanyuan Lu, Yanxia Wang, Daniel Mullan, John Hamblin, Chunji Liu

**Affiliations:** ^1^Faculty of Science, UWA School of Agriculture and Environment, University of Western Australia, Perth, WA, Australia; ^2^The UWA Institute of Agriculture, University of Western Australia, Crawley, WA, Australia; ^3^Hebei Centre of Plant Genetic Engineering, Institute of Genetics and Physiology, Hebei Academy of Agricultural and Forestry Sciences, Shijiazhuang, China; ^4^Inner Mongolia Academy of Agriculture and Animal Husbandry Sciences, Huhhot, China; ^5^Hebei Province Wheat Engineering Technical Research Center, Shijiazhuang Academy of Agricultural Sciences, Shijiazhuang, China; ^6^InterGrain Pty. Ltd., Bibra Lake, WA, Australia; ^7^SuperSeeds Technologies Pty. Ltd., Perth, WA, Australia; ^8^Commonwealth Scientific and Industrial Research Organisation Agriculture and Food, St. Lucia, QLD, Australia

**Keywords:** selfed pure lines, recombinant inbred lines (RILs), near isogenic lines (NILs), doubled haploid (DH), fast generation cycling system (FGCS), crop breeding

## Abstract

Production of pure lines is an important step in biological studies and breeding of many crop plants. The major types of pure lines for biological studies and breeding include doubled haploid (DH) lines, recombinant inbred lines (RILs), and near isogenic lines (NILs). DH lines can be produced through microspore and megaspore culture followed by chromosome doubling while RILs and NILs can be produced through introgressions or repeated selfing of hybrids. DH approach was developed as a quicker method than conventional method to produce pure lines. However, its drawbacks of genotype-dependency and only a single chance of recombination limited its wider application. A recently developed fast generation cycling system (FGCS) achieved similar times to those of DH for the production of selfed pure lines but is more versatile as it is much less genotype-dependent than DH technology and does not restrict recombination to a single event. The advantages and disadvantages of the technologies and their produced pure line populations for different purposes of biological research and breeding are discussed. The development of a concept of complete *in vitro* meiosis and mitosis system is also proposed. This could integrate with the recently developed technologies of single cell genomic sequencing and genome wide selection, leading to a complete laboratory based pre-breeding scheme.

## Introduction

High yield and quality with tolerance to biotic and abiotic stresses are the major breeding objectives of crop breeding and also of interest in genetic studies. Crop breeding programmes are frequently based on pure lines. These require 5–6 generations of inbreeding and selection after crossing to generate inbred homozygous lines suitable for extensive evaluation. This process is the reason for the time taken, often 11–13 years between making crosses to releasing cultivars. Similarly, the production of pure lines from segregating populations is also necessary to identify genes for a trait of interest.

The developed pure lines provide permanent populations allowing phenotyping of traits of the same genotypes at different times and under different environmental conditions. Both geneticists and breeders have been seeking ways to accelerate the process of pure line production. Doubled haploids (DH) are a successful technique for this purpose as it dramatically reduces the time for the production of homozygous pure lines. Since the first report of haploid production in 1920s, the DH, both the range of species and efficiency of production, has been greatly improved and is routinely used in many crop breeding programs (Asif, 2013; Li et al., 2013). Another system for pure line production is a fast generation cycling system (FGCS). This has been recently developed and applied in crop breeding (Wang et al., 1999, 2003; Ochatt et al., 2002; Zheng et al., 2013; Forster et al., 2014; Liu et al., 2016; Yao et al., 2016, 2017). FGCS shortens each generation cycle by culturing young embryos and managing plants to greatly reduce the time from seed to the seed of the next generation. The key difference between DH and FGCS technology lies in the greater opportunity for genetic recombination to occur in FGCS as it goes through more generations.

This review summarizes: (1) the general process of DH and FGCS technologies; (2) the applications of the produced pure line populations in genetic studies and crop breeding; (3) the constraints and opportunities of DH and FGCS technologies; and (4) the possible futures for technology improvement in fast development of pure lines.

## DH technology

**Figure 1:**
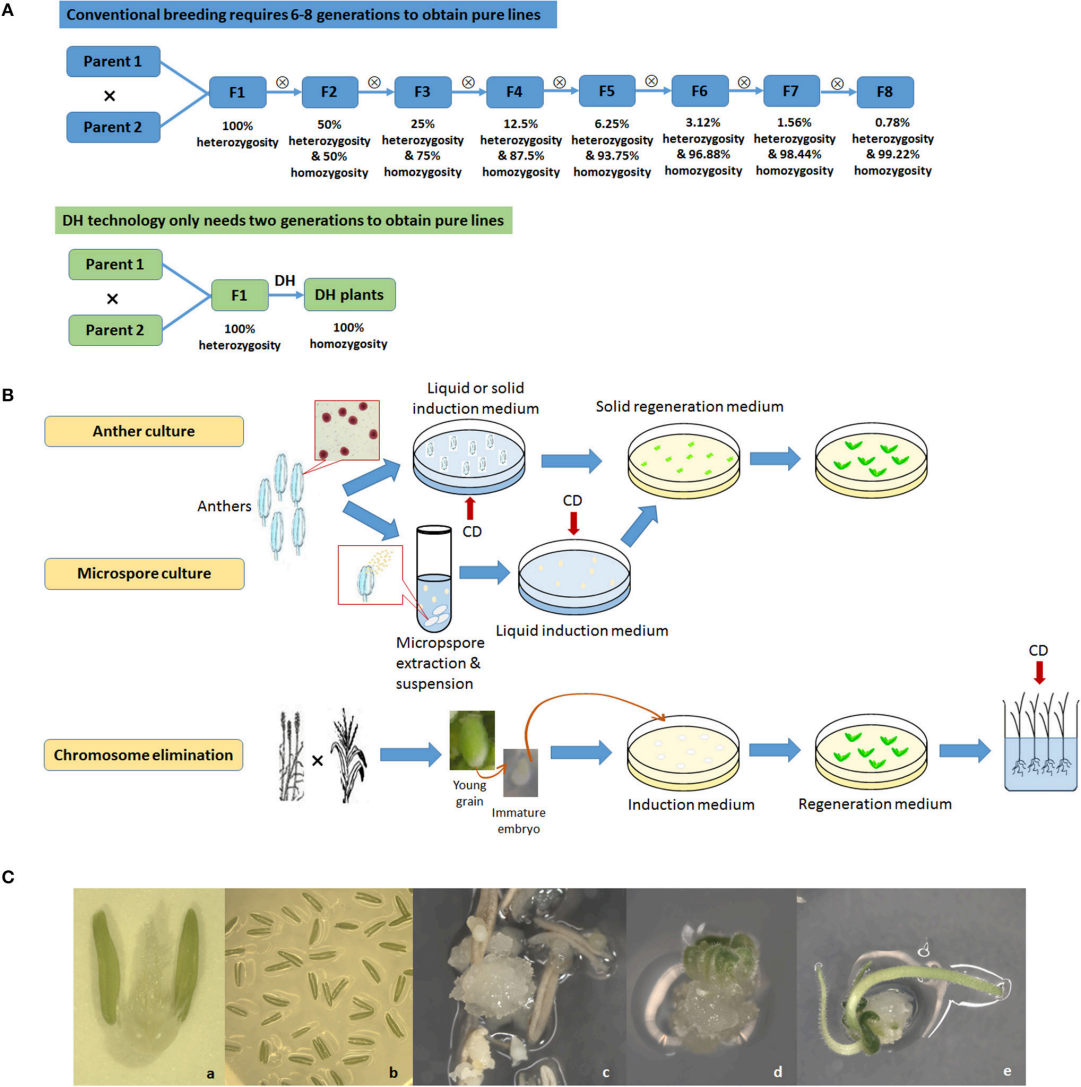
Doubled haploid (DH) technology. **(A)**, Comparison between conventional breeding and DH technology; **(B)**, Diagram of three major DH technologies adopted in crop breeding: anther culture, microspore culture and chromosome elimination. CD, Chromosome doubling with chemical treatment; **(C)**, Anther culture in wheat. a, Anthers on a flower; b, Anthers cultured on medium; c, Callus formation; d, Initiation of differentiation; e, Differentiation of roots and shoots.

DH generates homozygous lines by doubling chromosomes of haploid plants generated from either egg or sperm cells (Figure 1). There have been many reviews of DH technology in plants, covering a range of species, protocol improvements, recent concepts, and applications in genetic study and breeding (Maluszynski et al., 2003; Forster et al., 2007; Touraev et al., 2009; Dunwell, 2010; Forster and Thomas, 2010; Murovec and Bohanec, 2012; Asif, 2013; Dwivedi et al., 2015).

There are three widely used methods to produce DHs. androgenesis uses sperm cell microspore culture or another culture. Microspore culture uses microspores, preferably at the uni-nucleated stage, isolated from young anthers and then cultured *in vitro* where they develop into either embryoids or callus tissue that is regenerated into plantlets. Another culture uses developing anthers that are directly excised from flower buds at a critical stage and cultured *in vitro* where the microspores within the anthers develop into callus or embryoids that can be regenerated into plantlets. Chemical (usually colchicine) treatments for chromosome doubling may occur at either *in vitro* culture stage by using media containing the chemical to directly generate DH embryoids or callus or at a later stage on the regenerated haploid plantlets. The former is now the most often used methodology. The second method is gynogenesis, using ovary or ovule culture. The ovary or ovule is carefully dissected from the flower, a labor-intensive step that limits its wide adoption in crop breeding. The third method is chromosome elimination, where the target species is crossed to a distant related relative and the embryos produced are cultured or rescued *in vitro*. The chromosomes of the distant relative are eliminated, giving rise to haploid plantlets having only chromosomes from the target species (Jensen, 1983; Houben et al., 2011). The chromosomes of the regenerated haploid plantlets are then chemically doubled. Chromosome elimination has been successfully used in barley (*Hordeum vulgare*) crossed with *Hordeum bulbosum* (Kasha and Kao, 1970) and wheat (*Triticum aestivum*) crossed with maize (*Zea mays*; Laurie and Bennett, 1988; Inagaki and Tahir, 1990). In maize, a specific Stock-6 based intraspecific system (Coe, 1959) has been extensively exploited and has generated a majority of commercial DH maize cultivars. The genetic basis underlying the haploid induction in maize has been elucidated in recent studies (Hu et al., 2016; Gilles et al., 2017; Kelliher et al., 2017).

The advantages and disadvantages of these three methods and their optimizations have been extensively discussed (Reed, 2004; Bohanec, 2009; Germanà, 2011; Asif, 2013). Another culture is relatively easy to operate, and often quicker to produce DH lines compared to other methodologies as anther walls have positive effects on promoting culture growth. The disadvantage is that plantlets may originate from haploid microspore as well as from diploid anther wall tissue. Microspore culture eliminates this problem and is ideal for genetic manipulations as it starts from single cells that can be better regulated. Technological advances have now enabled microspore culture to achieve reasonably high yields in some crop genotypes, such as, canola, barley, and wheat (Gil-Humanes and Barro, 2009; Broughton et al., 2014). The efficiency of this technique, however, is highly genotype-dependent and its success rate may be low in many plant species (Cegielska-Taras et al., 2015). Chromosome elimination has an advantage of relatively easy laboratory protocols, but also has the issue of low efficiency. In addition, it requires an extra crossing procedure, and its chromosome doubling is usually applied to the regenerated haploid plants which involves direct contact with a large amount of hazardous chemicals.

New ideas for DH production have included centromere-mediated genome elimination to create a haploid inducer line (*GFPtailswap cenH3*^−/−^) in *Arabidopsis* (Ravi and Chan, 2010) and maize (Kelliher et al., 2016) for haploid production followed by chromosome doubling. Another method involving the use of unreduced gametes referred to “synthesizing DH (SynDH) technology” that employs interspecific hybridization between tetraploid and diploid lines followed by spontaneous polyploidization to obtain hexaploid plants has been described in wheat (Zhang et al., 2011).

The advantage of DH over conventional breeding methods is that DH achieves complete homozygosity in one generation. This enables significant shortening of time to the production of pure lines. Complete homozygosity allows more precise phenotyping and allows accurate gene-trait association in genetic mapping and gene function studies. The single cell cultures can also be used as targets for cell biology and genetic engineering studies. DH technology has been successfully developed and improved many crops (Table 1), in which barley and rapeseed are among the most responsive. However, there are constraints to the use of DH routinely for breeding and genetic study. Cottons and many legume species are recalcitrant to DH technology and despite major effort there have been few successes, while some others species suffer from high costs and low efficiency of DH technology. The major factors influencing the efficiency of DH production include species and genotype dependency (Ei-Hennawy et al., 2011; Murovec and Bohanec, 2012), a high proportion of albinism (Kumari et al., 2009; Makowska and Oleszczuk, 2014; Sriskandarajah et al., 2015), high frequencies of clones via androgenesis (Oleszczuk et al., 2014), and genome instability such as, aneuploidy due to somaclonal variation (Oleszczuk et al., 2011; Wedzony et al., 2015). These problems may jeopardize both breeding and genetic studies. In addition, DH requires skilled personnel and tissue culture facilities, which may not be available and DH only allows one or two opportunities of recombination, as DH lines are usually generated from F1 or sometimes F2 plants, limiting the diversity of the DH lines.

**Table 1:** DH technology in major crops.

**Crop names**	**Recent crop-specific reviews**	**Crop-specific protocols or technology improvements**
Barley (*Hordeum vulgare*)	Devaux and Kasha, 2009; Weyen, 2009; Houben et al., 2011	Broughton et al., 2014; Sriskandarajah et al., 2015
Wheat (*Triticum aestivum* and *Triticum turgidum* subsp. *durum*)	Weyen, 2009; Tadesse et al., 2013; Niu et al., 2014	Suenaga and Nakajima, 1989; Maluszynski et al., 2003; Kim and Baenziger, 2005; Broughton, 2011; Broughton et al., 2014
Rice (*Oryza sativa*)	Mishra et al., 2016	Maluszynski et al., 2003; Pauk et al., 2009
Maize (*Zea mays*)	Prasanna et al., 2012; Prigge and Melchinger, 2012	Maluszynski et al., 2003; CIMMYT, 2010; Prasanna et al., 2012
Oat (*Avena sativa*)	Basu et al., 2011	Kiviharju, 2009; Marcinska et al., 2013; Ferrie et al., 2014
Triticale (× Triticosecale)	Eudes and Chugh, 2009; Basu et al., 2011; Wedzony et al., 2015	Maluszynski et al., 2003; Asif et al., 2014a,b; Lantos et al., 2014
Rye (*Secale cereale*)	Basu et al., 2011	Maluszynski et al., 2003; Tenhola-Roininen et al., 2006
*Brassica* spp.	Ferrie and Mollers, 2011; Rahman and De Jimenez, 2016	Maluszynski et al., 2003; Gil-Humanes and Barro, 2009
Legumes (Fabaceae)	Croser et al., 2007; Lulsdorf et al., 2011	Ochatt et al., 2009; Lulsdorf et al., 2011
Fruit crops	Germanà, 2006	Germanà, 2006

## FGCS technology

**Figure 2:**
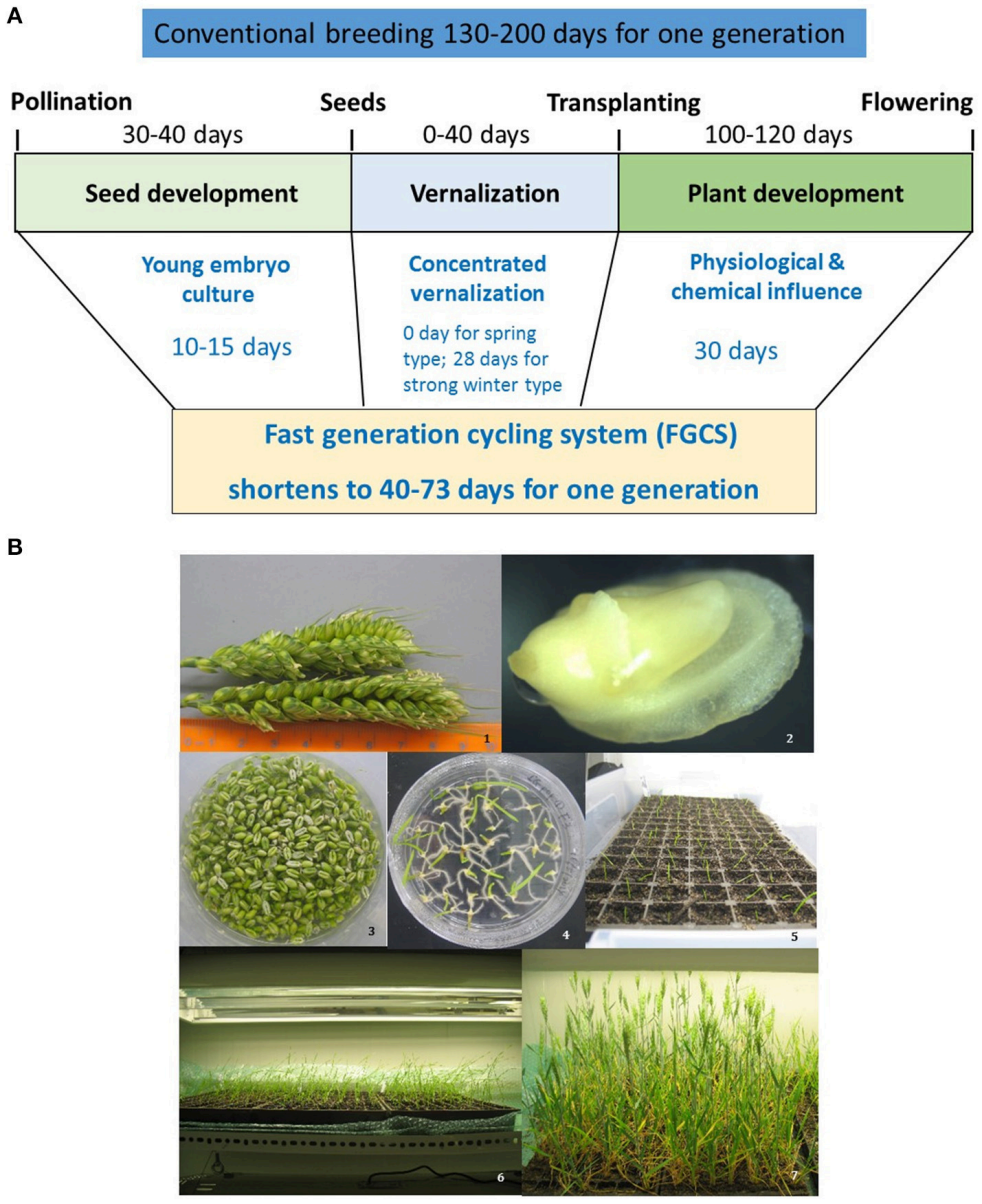
Fast generation cycling system (FGCS). **(A)**, Comparison of one generation time between conventional breeding and FGCS in crops. **(B)**, FGCS in wheat from young grains to flowering. 1, Yong wheat spikes collected for embryo culture; 2, An embryo dissected from a young wheat grain; 3, Young grains ready for embryo culture; 4, Germinated embryos in a plate; 5, Plantlets transferred to soil; 6, Plants grown into three-leaf stage; 7 Plants at flowering stage.

FGCS combines embryo culture with plant management to greatly reduce generation time (Figure 2). It involves two steps in each generation. Firstly, plants are grown in a controlled environment where irrigation and nutrient managements accelerate the vegetative growth and flower differentiation. Secondly, young embryos are cultured reducing the time required for seed maturation (Wang et al., 1999, 2003). At this step, the removing of endosperm promotes germination because of embryos' easy absorption of the readily available sucrose in the medium (Tezuka et al., 2012) and the detachment of possible inhibitors in the endosperm (Chawla, 2002). A fully *in vitro* protocol for FGCS has also been reported for some plants including protein legumes, *Arabidopsis* and wheat etc. (Ochatt et al., 2002; Ochatt and Sangwan, 2008, 2010; Yao et al., 2017).

Several advantages for FGCS have been reported: (1) it reduces the time from crossing to variety release as generation time for RILs and introgression are dramatically reduced; (2) it does not reduce the number of meiotic events where recombination occurs, providing the same recombination as traditional breeding procedures, which is beneficial for both crop breeding and high resolution genetic mapping or fine mapping studies; (3) selection can be incorporated in any generation and NILs can be developed using the heterogeneous inbred family (HIF) selection (Tuinstra et al., 1997); and (4) it is less genotype-dependent.

Factors influencing the efficiency of FGCS could exist in both embryo culture (Lentini et al., 1988; Raghavan, 2003) and plant management (Ausín et al., 2005; Riboni et al., 2014). Variation of embryo culture between crops occurs with embryo age and culture medium used (Table 2). The optimum embryo age for culture varies from as early as 7 days post anthesis (dpa) in rice to as late as 18–20 dpa in peas. The embryo culture medium generally contains basal salts (macro- and micro-nutrients), vitamins, sucrose, and medium solidifier, or supplemented with young coconut juice depending on crop species. As an energy source, sucrose plays an important role in physiology of seed germination. Young liquid endosperm of green coconuts is recognized as an important organic supplement for embryo culture in many plants (Molnár et al., 2011). It contains a number of amino acids, organic acids, nucleic acids, vitamins, plant hormones, sugar alcohols, and some unidentified substances that may be responsible for promoting embryo growth. Environmental factors affecting vegetative growth and flower differentiation include light, temperature, irrigation, and nutrient solutions. Optimizing these are crucial in determining the efficiency of FGCS (Whitman et al., 1998; Cerdán and Chory, 2003; Cummings et al., 2007). Environmental stress may also accelerate flowering (Kumar et al., 2012). Warm temperatures and/or moderate drought can act as environmental cue to flowering in some plants (Riboni et al., 2014). Increased light intensity or photoperiod duration (such as, constant light) accelerate flowering in *Arabidopsis* (Kumar et al., 2012). Extreme examples of environmental stress leading to fast flowering occurs in some desert annual plants with no juvenile stage. Atmospheric CO_2_ concentration and tiller removal also affect FGCS in rice (Ohnishi et al., 2011). For soil nutrients, nitrogen usually delays reproduction and phosphorous and potassium usually promote reproduction (Wang et al., 2003). It has also been reported that small pot size with limited nutrient supply and limited irrigation prevent tiller production in cereal plants, shortening the vegetative period thus promoting flowering (Forster et al., 2014). Shortening the juvenile phase may result in early maturity (soybean and rice). The duration of juvenility can influence flowering regardless of other physiological controls of flowering (Atwell et al., 1999). To speed up the generation cycle, a balance between short and sufficient vegetative growth is needed to achieve development but must be balanced with enough biomass accumulation for flower initiation and seed set.

**Table 2:** FGCS technology in major crops.

**Crop name**	**Embryo age ready for culture (days post anthesis)**	**Embryo culture medium**	**One generation time using FGCS (days)**	**Generations per year using FGCS**	**References**
Barley (*Hordeum vulgare*)	>10 (optimum 12)	MS (salts + vitamins) + 1% sucrose + 10% young coconut juice + 0.52% phytagel (pH 5.8)	39–50	Up to 9 generations per year	Zheng et al., 2013
Wheat (*Triticum aestivum*)	>10 (optimum 12)	MS (salts + vitamins) + 1% sucrose + 10% young coconut juice + 0.52% phytagel (pH 5.8)	42–55	Up to 8 generations per year	Wang et al., 2003; Zheng et al., 2013; Yao et al., 2017
Rice (*Oryza sativa*)	7	$\frac{1}{2}$ MS salts + 2% sucrose + 0.8% agar (pH 5.8)	60	6 generations per year	Ohnishi et al., 2011
Maize (*Zea mays*)			60	6 generations per year	Pioneer, 2008
Oat (*Avena sativa*)	12	MS (salts + vitamins) + 1% sucrose + 10% young coconut juice + 0.52% phytagel (pH 5.8)	49–59	Up to 7 generations per year	Liu et al., 2016
Triticale (× Triticosecale)	12	MS (salts + vitamins) + 1% sucrose + 10% young coconut juice + 0.52% phytagel (pH 5.8)	48–61	Up to 7 generations per year	Liu et al., 2016
*Brassica* spp.	10–12	MS (salts + vitamins) + 1% sucrose + 10% young coconut juice + 0.52% phytagel (pH 5.8)	48–56	Up to 7 generations per year in *B. napus*	Yao et al., 2016
Legumes (Fabaceae)	18–20 in pea (*Pisum sativum*)	MS (salts + vitamins) + 0.6% agar (pH5.6 – 6) (with or without sucrose or growth regulators according to species)	50–90 (pea)	5–6 generations per year (pea)	Ochatt and Sangwan, 2010; Ribalta et al., 2014

Other factors affecting FGCS include seed dormancy and vernalization. These affect seed/embryo germination and plant flowering, respectively. Embryo age has an important role in the efficiency of embryo culture where uniform and rapid germination is ideal but may be staggered by the effects of seed dormancy (Lefebvre et al., 2006; Hilhorst, 2007; Lee et al., 2010; Kang et al., 2015).

Early embryo culture can be carried out without waiting for full seed development and embryos can be harvested before dormancy sets in. When young embryos are dissected at a stage suitable for culturing on artificial medium, this could effectively circumvent seed dormancy. Although exactly when dormancy develops during seed development is unclear, it is considered to be initiated at the stage when food reserves start to accumulate, which is after the embryo develops into the heart stage (Bentsink and Koornneef, 2008). In cereal crops, seed dormancy could develop before the hard dough stage, as reported in wheat (Lan et al., 2005). Among the different types of seed dormancy, non-deep physiological dormancy is common in many crop species. It is determined by physiological factors in the embryo and surrounding layers including the endosperm and the testa. Embryos excised from seeds with such dormancy will usually germinate normally and treatments of gibberellins (GA), scarification, stratification, or a period of dry storage can break the dormancy (Atwell et al., 1999; Linkies et al., 2010; Willis et al., 2014).

Long season winter crops are important but their generation time is long due to their requirement for vernalization. These crops require 25 to at least 40 days of vernalization. This limits the efficiency of marker development and trait recombination in these crops. FGCS can be applied to winter crops by vernalizing germinated embryos before transplanting them into soil. With this procedure up to five generations of winter wheat per year have been achieved (Wang et al., 1999, 2003). In our study applying FGCS in winter wheat it was possible to reduce generation time by bypassing the vernalization requirement. Vernalization promotes flowering by causing an epigenetic shut off of *FLOWERING LOCUS C* (*FLC*) gene coding for a repressor of flowering. The status of the epigenetic modification can be changed. This is known as “memory” and “resetting,” with the former referring to the mitotic memory system of the histone modifications triggered by vernalization. This allows flowering to occur and reprogrammes the epigenetic states to their default position in the next generation, ensuring flowering occurs at an optimal time in every generation (Sheldon et al., 2008; Choi et al., 2009; Dennis and Peacock, 2009; Oliver et al., 2009; Crevillén et al., 2014; Woods, 2014). When FGCS was applied to winter wheat varieties, it was found that older embryos (more than 20 dpa) required vernalization to initiate flowering whereas plants generated from younger embryos (15 dpa) flowered without vernalization (Qin and Wang, 2002). The memory of the vernalized state may be maintained in the young embryos before the resetting of the system as the embryos aged. Screening a wide range of winter wheat varieties is needed in future studies so that the optimum age of embryo development can be determined to ensure that the generated plants flower without vernalization.

FGCS plants can be grown entirely in soil or *in vitro* as found in FGCS of protein legume crops (Ochatt et al., 2002). The earliest use of FGCS entirely in soil was in *Brassica* where rapid flowering species were used and grown at high densities in a controlled environment (constant temperature and light conditions) allowing up to 10 generations per year in *B. rapa* (Williams and Hill, 1986). Entirely *in vitro* FGCS is to complete a generation cycle from dissecting and germinating young embryo to inducing flowering and seed set totally under *in vitro* conditions, which is repeated until the desired generation (Yao et al., 2016). Plant growth regulators are sometimes needed for this system, as reported in *Pisum* and *Vigna* (Ochatt and Sangwan, 2008; Ribalta et al., 2014).

FGCS is cost effective as it saves time and space and is comparable to DH in regards of achieving pure lines with desired homozygosity. Single seed descent (SSD) is usually adopted in FGCS for developing RIL populations in crops, through continuous selfing from the F_2_ generation until the desired level of homozygosity is reached. For high resolution genetic analysis, a large F_2_ population is needed to ensure that later generation population size is representative and suitable for any statistically sound genetic analysis. Therefore, F_1_ plants must be grown under optimum conditions ensuring the maximum number of F_2_ seeds or embryos before FGCS can be applied. DH also require optimum conditions for the F_1_ plants to produce large numbers of healthy anthers/microspores/ovaries needed for DH production. The time taken for growing F_1_ plants is therefore similar for both DH and FGCS. From F_1_ flowering stage, production of DH lines can be achieved within a year or one and half years for many crops, whereas the time taken for FGCS to produce RILs (F_6_ or F_8_ depending on required use) from F_1_ embryos can also be achieved within a similar timeframe in the same crops (Liu et al., 2016). In FGCS, plants are grown in small pots or tray cells which require significantly less space and labor cost for large population development than that of the field conditions in conventional breeding. Being less genotype-dependent as shown in previous studies (Liu et al., 2016; Yao et al., 2016), FGCS can also provide savings compared to DH in crops where recalcitrant genotypes occur, for example, some oat genotypes having low DH efficiency with low haploid embryo production rates and haploid plant regeneration rates needed to be compensated by plating more anthers/microspores or embryos to raise the yield of DH production, which increased the labor and cost (Kiviharju et al., 2005; Marcinska et al., 2013).

FGCS is especially attractive in species or genotypes where DH lines are difficult to produce. Successful applications of FGCS were reported in several major crops, which significantly shortened the generation time and enabled 6–9 generations per year in the crops that would otherwise only allowed up to 1–3 generations per year by conventional methods (Table 2).

## Application of pure line populations in genetic studies and breeding

Unlike early generation segregation populations such as, F_2_ and backcross lines, DH lines, RILs, and NILs are pure line populations because they are homozygous that can be multiplied and reproduced without genetic changes occurring (Semagn et al., 2006). They can be repeatedly used in trials, which is important for estimates of trait heritability and for genetic analyses such as, mapping and gene function studies. Each of the approaches has advantages and disadvantages when used in genetic studies and breeding.

## DH lines

DH lines are complete homozygotes and contain two identical sets of chromosomes/genes. They are ideal for estimating quantitative trait locus (QTL) × environment (E) interactions as complete homozygosity allows better estimates of trait means and allows more precise selection over locations and years. The expected genotypic segregation ratio is 1:1, irrespective of whether the marker is dominant or codominant. DH plants are fully fertile and if suitable can be used as parents or released as a cultivar by breeding programs. DHs have been widely used for cultivar development, genetic mapping, mutagenesis, and gene function studies (Ferrie and Mollers, 2011; Hussain et al., 2012).

However, distorted segregation ratios can be observed, reducing the accuracy of genetic maps. This may be due to several causes: (1) genetic factors due to gametic or zygotic selection for pollen tube competition, preferential fertilization, chromosome translocation, etc. (Liu et al., 2010); (2) the genotype-dependency of DH, i.e., the different responses of the cross parents to DH method (Tanhuanpää et al., 2008); (3) somaclonal variation arising during DH production resulting in aneuploid production (Oleszczuk et al., 2011); and (4) high frequencies of clones via androgenesis (Oleszczuk et al., 2014).

Haploids have long been proved to be invaluable materials for basic genetic studies and they can be used for quick generation of quadruple, quintuple, sextuple, or higher order multiple mutant combinations, production of homozygous maternal gametophyte lethal mutants, and detection of gene conversion events during meiosis. (Wijnker et al., 2013; Ravi et al., 2014; Fulchar and Riha, 2016). A new breeding concept “reverse breeding” using DH has recently been proposed and successfully demonstrated in *Arabidopsis*, in which the approach also enabled the quick generation of a series of chromosome substitution lines (Wijnker et al., 2012).

DH lines produced from chromosome doubling of pollen/egg derived haploid plants of F_1_ plants only have one recombination opportunity in the first generation. To increase recombination, sometime F_2_ pollen/egg is used instead of F_1_'s for haploid production. The costs involved in establishing a reliable protocol for a wide range of genotypes is also a limiting factor restricting the general application of DH to crop breeding. The centromere mediated genome elimination method reported in *Arabidopsis* and maize (Ravi and Chan, 2010; Kelliher et al., 2016) is particularly attractive because it is easy to adopt (only one crossing of the target genotype with the haploid inducer line is needed) and it produces haploid seeds without the necessity of embryo rescue (Ravi et al., 2014). Although it has not yet been successfully employed in other species probably due to the lack of *cenH3* knockouts, the recent development of CRISPR-based genome edition targeting specific genes might be the solution to finally translate this technology into other crops (Britt and Kuppu, 2016).

## RILs

RILs are the products of successive inbreeding over several generations to develop true breeding lines. In self-pollinated crops, it is usually achieved by a SSD approach which involves continuous selfing of individuals from an initial F_2_ population until the desired level of homozygosity is achieved. F_8_ RILs are often used for genetic studies, where 99% homozygosity is expected (Seymour et al., 2012). As a pure line population, RILs can also be replicated over locations and years and shared among different research groups. As with DHs, the expected genetic segregation ratio of RILs and the overall frequency of alleles for both dominant and codominant marker are 1:1. RILs are often used to map traits that differ between the parental lines. As a product of many meiotic cycles, RILs are expected to have higher recombination rate than DH, which is not only important for QTL mapping but also very useful to identify tightly linked markers. RILs are difficult to develop in crops that exhibit high levels of inbreeding depression (Madhusudhana, 2015). For these cross-pollinated crops, RIL development can be achieved by sib-mating but takes longer due to the many crosses involved. Being nearly homozygous, RILs are similar to DH lines and can be used to estimate QTL × E interaction and additive and epistatic effects of multiple loci contributions to trait expressions, but not dominant effects that require analysis of segregating populations such as, F_2_ or early backcross populations.

Given the fact that RILs are not complete homozygotes and each RIL may harbor confounding genetic variation with the threat of residual recombination in further generations, they may be less statistically powerful than DH for analyzing effects of particular loci. Distorted segregation can also be observed in RILs due to genetic factors resulting in gametic and zygotic selection (Liu et al., 2010).

Several studies compared DH and RIL populations for linkage map construction and QTL analysis. Somers and Humphreys (2009) found that wheat RILs had nearly twice the number of breakpoints per chromosome compared to DHs. This is consistent with theoretical expectation as RILs pass through more meiotic events in development and should have greater recombination creating more breakpoints along the chromosomes. Sorrells et al. (2011) also reported that the wheat reference population SynOpRILs showed a higher frequency of segregation than the SynOpDH lines. Zhao et al. (2010, 2013) observed significantly larger transgressive segregations for most agronomic traits, and higher QTL × E interactions in RIL compared to DH populations. The common thread in these studies is that the QTLs identified for the same trait are mostly different by using DH and RIL populations, even when the DHs and RILs were derived from the same cross. This was attributed to the quantitative traits being controlled by large numbers of QTLs that were sensitive to environmental effects (Zhao et al., 2013).

In Australia, cereals, oilseeds, and grain legumes are produced on a large scale for human consumption and livestock feed. Most of these cultivated crops are spring-type and self-pollinated that are especially suitable for developing RILs using FGCS.

## NILs

**Figure 3:**
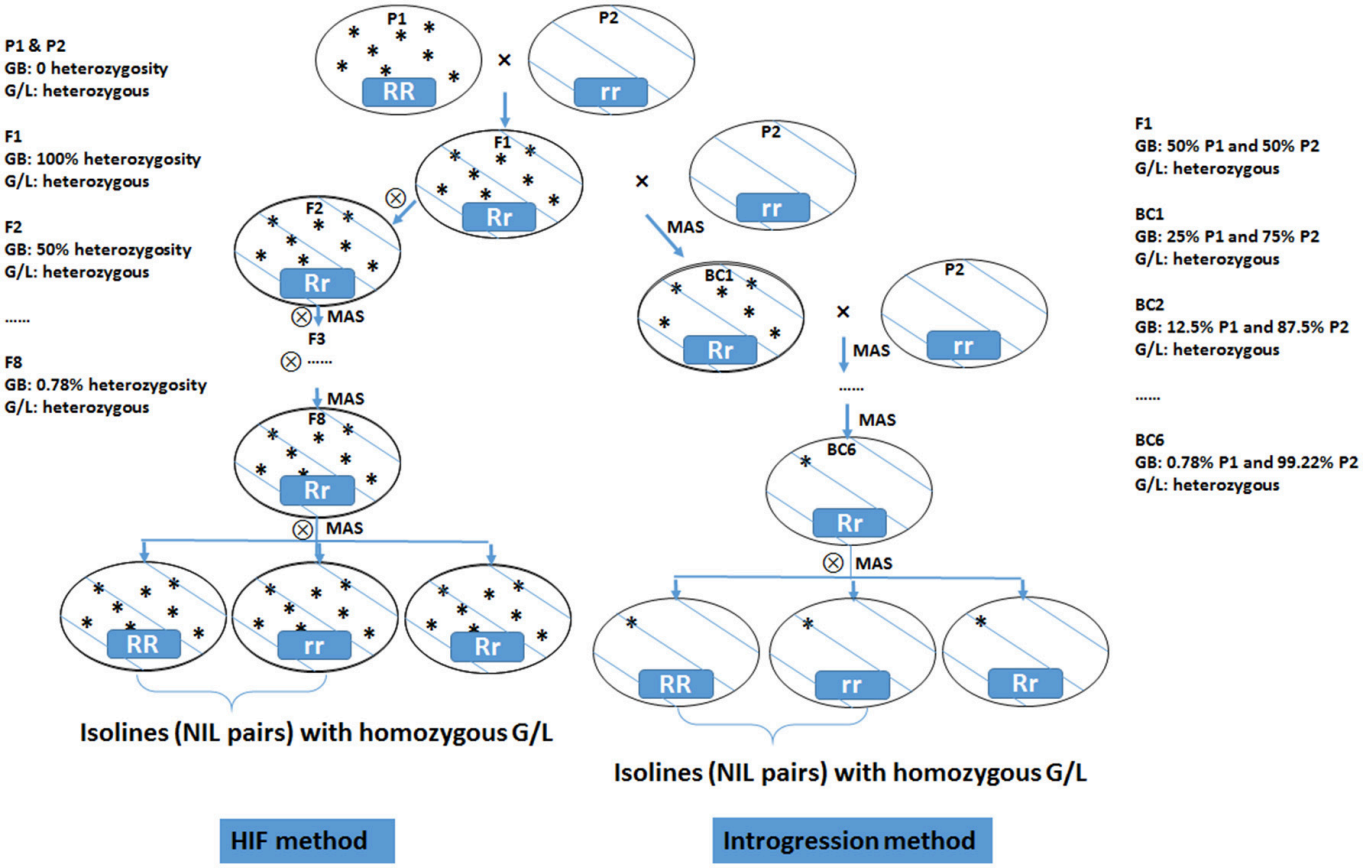
Diagram of heterogeneous inbred family (HIF) method and introgression method for developing near isogenic lines (NILs) from two inbred parental lines. GB, genetic background; G/L, target gene/locus, with RR representing dominant allele and rr representing recessive allele; MAS, marker assisted selection.

NILs are pairs of lines having identical genetic background but contrasting at the targeted genomic region. NIL iso-lines segregate primarily on the targeted locus/gene while all other genes affecting the trait of interest are the same. Thus, any phenotypic difference between the iso-lines can be assigned to the targeted locus/gene. This allows the conversion of a quantitative trait into a Mendelian factor, making detailed mapping and gene discovery possible. NILs can be developed by repeated selfing using the HIF method (Tuinstra et al., 1997) or by repeated backcrossing (Figure 3). An HIF population is developed from a cross between two inbred lines. A progeny with target heterozygous locus is chosen by marker selection in F_2_ population. This is selfed and then from F_3_ on, the progenies that are heterozygous at the target locus are selected in each generation. By these processes, the genetic background becomes homozygous whereas the desired locus remains heterozygous. In the F_8_ generation, the heterozygotes are selfed to produce two NILs that are homozygous and contrasting (either positive or negative) at the targeted locus (Ma et al., 2012; Habib et al., 2016).

Using a backcross approach with marker selection for the trait of interest introgressed from the donor parent, it is possible to isolate the introgressed character from the donor genome in the recurrent parent. The introgression line is then selfed to generate the NIL pairs with or without the targeted locus. When screening with molecular markers, NILs are only polymorphic to the locus of interest.

NILs allow allelic variation at a targeted locus to be assessed enabling a detailed study of specific gene action at the physiological and developmental level (Moeller et al., 2014; Farré et al., 2016). Tagging traits are essential for functional genomics studies. NILs can also be used for the fine mapping of a gene, paving the way for gene isolation using positional cloning and allowing the development of tightly-linked genetic markers. The comparison of mRNA and protein expression in the NIL pairs can lead to the identification of candidate genes. If available, differentially expressed genes can be sequenced and mapped to a reference genome, to determine whether they are within a particular QTL chromosomal region (Ma et al., 2014; Barrero et al., 2015; Brechenmacher et al., 2015).

Preliminary QTL mapping by using DH or RIL populations has only limited resolution. Markers obtained from such studies may not be reliably used in tagging the targeted loci. Variations in genetic backgrounds of the mapping populations may interfere with the accurate phenotyping of a targeted trait. Thus, lines containing the same targeted allele may exhibit different phenotypes (Zheng et al., 2015). NIL derived fine mapping populations with fixed genetic backgrounds, except for the targeted genomic region, are suitable for gene discovery and for developing tightly linked markers. Fine mapping populations can be derived from NILs using heterozygous lines in advanced generations (for example, F_8_ or BC_7_) during development of the NILs. Usually a single heterozygous plant is used to be further advanced two or three more generations, to generate a large population allowing more recombination events within the targeted QTL region. The fine mapping population can then be phenotyped to establish the presence or absence of the QTL in that particular region, and ultimately reduce the QTL to a single or a few genes which can then be tested using functional genomics (Kooke et al., 2012; Zheng et al., 2015).

Like RILs, NILs require many generations to develop. Using FGCS can reduce the time needed for this. In FGCS, a moderate environmental stress level is used to promote reproduction whilst at the same time allowing enough vegetative growth for seed production. The volume of potting mix, temperature, and light conditions all affect the stress level and therefore affect the durations of the plant generation cycle as well as the number of seeds produced per plant. For NIL development, a larger number of progenies in each generation is required than is the case for RIL where only one seed is needed, hence a slightly larger potting soil volume is necessary to produce more seeds for each line, allowing selection for heterozygous or homozygous lines from them. Although this reduces stress level which delays flowering, a reasonably short generation cycle can still be achieved using the FGCS (Zheng et al., 2013).

NILs can only be used to study one locus at a time. The candidate locus typically comes from preliminary QTL mapping studies by using RILs or DH lines (Table 3). For crops with a large genome size, such as, wheat, many generations are needed to advance the lines to near-homozygosity allowing precise genetic mapping of the gene. Linkage drag, the simultaneous transfer of closely linked undesired genetic factor together with the targeted locus, can be a potential problem in the development of NILs (Madhusudhana, 2015).

**Table 3:** Applicability of pure line populations in genetic studies and breeding.

**Population type**	**Generating new breeding material**	**Preliminary QTL mapping**	**Fine mapping**	**Gene identification**	**Gene function analysis**
DH lines	Yes	Yes	No	No	Yes
RILs	Yes	Yes	No	No	No
NILs	No	No	Yes	Yes	Yes

## Potential new approaches

Fast development of pure lines using DH or FGCS can be improved. In DH, genotype-dependency for haploid production is a major obstacle for its wide application. Research aimed at achieving DH production independent of the genotypes will improve the efficiency of the technology. The synthesizing DH methodology is only suitable for (allo)polyploids that can produce viable unreduced restitution gametes (Zhang et al., 2011). The CenH3 based haploid production system reported in *Arabidopsis* and maize (Ravi and Chan, 2010; Kelliher et al., 2016) is promising, but has not yet been successfully used in other crops.

De La Fuente et al. (2013) proposed a concept of rapid generation cycling of meiosis and mitosis. The procedure uses plant cell culture system. Single somatic cells are induced to undergo meiosis to generate gametes which can be artificially genome-doubled to produce DH lines; or the gametes can be fused to form diploid cells which, equivalent to a zygote, can be regenerated to produce plants or be induced again to undergo new meiosis to start a new generation cycle. The procedure can be repeated for many generations in an *in vitro* system. Marker assisted genomic selection will be needed for evaluation and selection of the gametes or zygotes in each generation. The system, if successful, allows increased efficiency of pure line production and genetic introgression. It not only shortens each generation cycle, but also eliminates the process of multi-generation crosses. Major technical challenges involved in developing such a system include somatic meiosis induction, cell fusion, and regeneration plants from single cells. Although somatic meiosis (Yoshida and Yamaguchi, 1973; Chen et al., 2000, 2001), gamete fusion (Faure et al., 1994; Ponya et al., 2004; Uchiumi et al., 2007; Kranz and Scholten, 2008) and single cell regeneration (Vasil, 1983; Yang et al., 1991; Birnbaum and Sánchez Alvarado, 2008; Ikeuchi et al., 2013) have been reported in plants, there are few reports of a single crop species undergoing all these processes successfully. In addition, the efficiency (how quickly and how many normal gametes or zygotes can be produced) of each step remains unclear. Therefore, the concept of *in vitro* meiosis and mitosis has not yet realized in practice.

**Figure 4:**
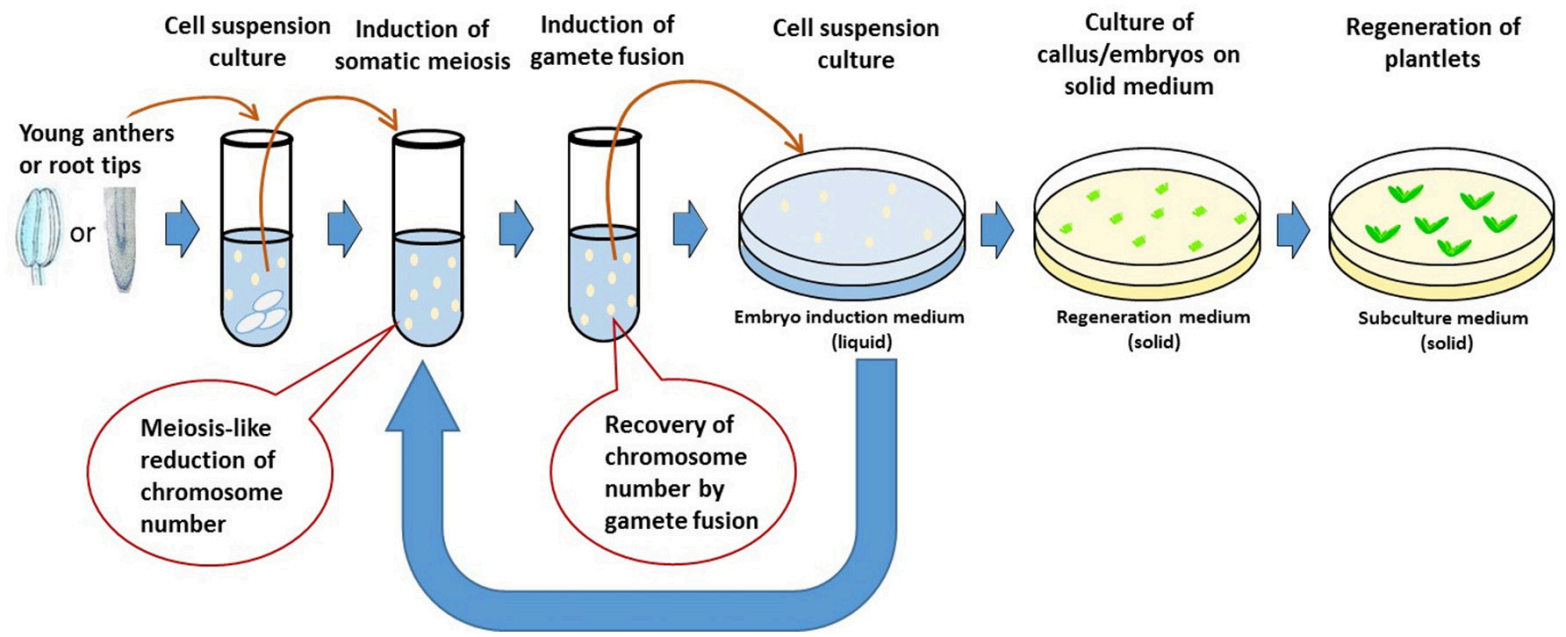
Diagram showing the concept of *in vitro* meiosis and mitosis system.

Interestingly, each of the necessary steps involved in the *in vitro* meiosis and mitosis system (IVMMS) has been reported as successful in wheat, for example, cell culture in suspension (Yang et al., 1991; Biesaga-Kos'Cielniak et al., 2008; Dong et al., 2010), somatic meiosis (Knott, 1956), gamete fusion (Kranz and Lorz, 1993; Zhou et al., 2002; Ge et al., 2006) and regeneration from somatic zygotes (Li et al., 1992; Kovács et al., 1995; Xia and Chen, 1996), which may be integrated to realize the concept of IVMMS in the crop (Figure 4). As somatic meiosis was reported mostly in either root tip cells (Mehar and Dhiman, 1986) or pre-meiotic cells (Knott, 1956), root tip cells and pollen mother cells can be used and treated with different combinations of somatic meiosis inducers (e.g., purine or pyrimidine derivatives or chloramphenicol) to induce somatic meiosis. The resulting cell culture can then be treated [e.g., with polyethylene glycol (PEG) or electric shock] to induce somatic fertilization. The fused cells may either be regenerated to plants or be induced again and again to complete more generations of meiosis and mitosis before regeneration to pure line plants. Cytogenetic studies at each of the step allows chromosome number of the cells to be determined, thus identifying the efficiency of somatic meiosis and somatic fertilization.

The IVMMS technology, if successful, can be integrated into breeding programs and make a new step-change for accelerating the breeding process. The plants resulting from IVMMS are expected to go through the similar recombination events, thus will have similar diversity, as those developed from conventional breeding. Along with the advent of new technologies, such as, single cell genomic sequencing (Macaulay and Voet, 2014) and genome wide selection (Goddard and Hayes, 2007; Morrell et al., 2012), IVMMS may allow a complete laboratory pre-breeding scheme to be established in major crops. Genome-wide selection is advantageous for predicting breeding value with high accuracy, as variation in most important traits are determined by many genes. Genomic sequencing of single cell progenies produced in IVMMS will allow the selection to be done completely *in vitro*, without the need to grow the plant lines. The IVMMS technology could also shed light into plant evolution. Genome-wide sequencing in the resulting progenies from IVMMS could reveal details about the evolutionary mechanisms in the plants that drive genetic divergence in the progenies that share a common original parent.

High density marker maps have been developed in some crops (Li et al., 2016; Punnuri et al., 2016), which makes genomic selection possible. Together with fast development of pure lines, the efficiency of crop genetic studies and breeding are expected to be significantly improved in the future.

## Author contributions

GY and HL conceived the ideas and developed the draft. HW, ZL, YW, DM, JH, and CL commented and revised the manuscript.

### Conflict of interest statement

The authors declare that the research was conducted in the absence of any commercial or financial relationships that could be construed as a potential conflict of interest.
